# Limitations of Personalized Medicine and Gene Assays for Breast Cancer

**DOI:** 10.7759/cureus.1100

**Published:** 2017-03-17

**Authors:** David Tiberi, Laura Masucci, Daniel Shedid, Isabelle Roy, Toni Vu, Erica Patocskai, André Robidoux, Philip Wong

**Affiliations:** 1 Department of Radiation Oncology, Centre Hospitalier de l'Université de Montréal (CHUM); 2 Department of Surgery-Neurosurgery Service, Centre Hospitalier de l'Université de Montréal (CHUM); 3 Department of Radiation Oncology, Cité De La Santé De Montréal, Université de Montréal; 4 Department of Surgery, Centre Hospitalier De L'université De Montréal (CHUM) Notre Dame; 5 Department of Surgery, Centre Hospitalier De L'université De Montréal (CHUM) Notre Dame

**Keywords:** breast cancer, neuroendocrine differentiation, gene assays, genomics, personalized medicine, oncotype dx

## Abstract

Adjuvant systemic treatments reduce the risk of breast cancer recurrence following the local treatment of primary stage I-III breast cancers. For patients with hormone-positive breast cancers receiving hormonal therapy, the risk of distant recurrence is under 20% and therefore, many patients may potentially be spared of chemotherapy. Consequently, several molecular signatures based on gene expression were developed to better determine which breast cancer patients would benefit from chemotherapy.

We present the case of a 62-year-old woman diagnosed with an early stage hormone receptor-positive breast cancer that was treated with a partial mastectomy. Oncotype DX (Genomic Health, Redwood City, CA) molecular testing was performed on the surgical specimen, which reported a recurrence score of 0. The patient commenced adjuvant radiotherapy during which she developed symptoms suggestive of bone metastasis and was subsequently diagnosed with a spinal cord compression that required neurosurgery and radiotherapy. Pathology review of the specimen from the spine surgery revealed a metastatic breast carcinoma with neuroendocrine differentiation.

Molecular assays such as Oncotype DX are increasingly used to prognosticate patient outcomes and help determine who may avoid chemotherapy. This case report seeks to illustrate that such assays should not be used in the presence of rare histological subtypes like neuroendocrine breast cancers, which are often under-reported. The current status of personalized medicine and gene assays in breast cancer is reviewed and potential strategies are suggested to identify these rare cases to better orient diagnostic and treatment decisions.

## Introduction

Adjuvant systemic treatments reduce the risk of breast cancer recurrence following the local treatment of primary stage I-III breast cancers. In women diagnosed with estrogen receptor (ER)-positive breast cancers, endocrine therapy such as tamoxifen reduces the annual breast cancer death rate by 31% which is largely irrespective of the use of chemotherapy, patient age, or tumor characteristics [1]. Similar analyses suggested that anthracycline-based chemotherapies reduce the risk ratio for any recurrence at 10 years to 0.73, which represents an 8% absolute reduction in the recurrences percentage [2]. The absolute likelihood of distant recurrence in patients receiving tamoxifen after surgery is less than 20% at 10 years [3]. Therefore, many patients may potentially be spared of chemotherapy. Consequently, several molecular signatures based on gene expression were developed to better determine which breast cancer patients would benefit from chemotherapy.

The Oncotype DX assay (Genomic Health, Redwood City, CA) is a molecular assay developed for node-negative, ER-positive breast cancer patients. The assay consists in quantifying the expression of 21-genes from formalin fixed paraffin embedded materials that is then converted into a recurrence score (RS) [4]. The RS is an independent predictor of distant recurrences in patients receiving endocrine therapy and thereby estimates the potential benefit of adding adjuvant chemotherapy in the patient’s cancer care. 

The clinical utility of the Oncotype DX assay was examined in several large studies. The first study validated the prognostic ability of the RS by retrospectively classifying 668 NSABP-B14 trial patients into low, intermediate or high-risk groups of developing distant recurrences among node-negative, ER-positive patients on tamoxifen [4]. A subsequent study demonstrated that the magnitude of benefit from adjuvant chemotherapy is the largest among high-risk patients (RS > 30) (relative risk, 0.26; 95% CI, 0.13 to 0.53) and smallest among patients classified at low risk (RS < 18) (relative risk, 1.31; 95% CI, 0.46 to 3.78) [5]; suggesting that RS is a potential predictive biomarker of adjuvant chemotherapy.

The trial assigning individualized options for treatment (TAILORx) is a prospective validation study that sought to assess the clinical utility of the Oncotype DX assay in node-negative, ER-positive breast cancer patients [6]. In this study, patients were conservatively stratified such that those with RS 0-10 were classified as low-risk patients and received hormone therapy alone. Patients with RS > 26 received chemotherapy and those with RS 11-25 were randomly assigned to receive endocrine therapy with or without chemotherapy. Results from this study’s low-risk cohort (N=1626) showed that the five-year rate of freedom from distant recurrences was 99.3% (95% CI, 98.7-99.6), in which 10 distant recurrences were detected. The study thus concluded that node-negative, ER-positive and human epidermal factor receptor 2 (HER-2) negative breast cancer patients with a low RS may be spared of adjuvant chemotherapy [6]. 

## Case presentation

We present the case of a 62-year-old woman who was diagnosed with breast cancer following an abnormal screening mammography in 2015. The patient’s past medical history is remarkable for Lynch syndrome, an autosomal dominant disease characterized by germline mutations of mismatched repair genes, initially diagnosed in 1990 for which the patient was regularly followed up in genetic medicine with serial pelvic ultrasound, cell surface antigen (CA-125), and colonoscopy every 18 months.

The initial workup consisting of a mammography and ultrasound imaging of the breast revealed a right-sided asymmetric density measuring approximately 16 mm. A stereotactic biopsy was performed and confirmed a grade 2 invasive ductal carcinoma that expressed both the ER and progesterone receptors (PR). The HER-2 status was negative as determined by fluorescence in-situ hybridization (FISH). No other staging exams (such as a bone scan) were performed since the patient was otherwise asymptomatic at initial presentation.

The patient underwent a right partial mastectomy with sentinel node sampling two months after her initial biopsy. The final surgical pathology report described a 22 mm grade 2, node-negative, ER/PR positive, HER-2 negative invasive ductal carcinoma. The surgical margins were negative and no perineural or lymphovascular invasion was identified.

A sample of the surgical specimen was sent for Oncotype DX testing, which reported an RS of 0, suggesting that this patient was at low risk of developing distant metastases. Thus, the patient was not offered adjuvant chemotherapy and was prescribed letrozole. She started adjuvant whole-breast radiotherapy (42.5 Gy in 16 fractions) at four weeks post-operatively.

After 8 fractions of radiotherapy, the patient developed acute dorso-lumbar pain and eventually urinary retention. Magnetic resonance imaging (MRI) of the dorso-lumbar spine was performed two weeks after radiotherapy which revealed three lytic lesions compatible with bone metastases at the right iliac bone, L3 and S2-S3 with soft-tissue epidural extension compressing the S2-S3 nerve roots. The patient underwent minimally invasive surgery with excision of the S2-S3 lesion followed by postoperative stereotactic body radiation therapy (SBRT) to a dose of 24 Gy in 3 fractions to the L3 lesion, and 35 Gy in 5 fractions to the other two sites. The pathology report of the decompressive surgical specimen was consistent with the diagnosis of a metastatic breast carcinoma with neuroendocrine differentiation as the cells expressed ER, PR, chromogranin A and synaptophysin. Upon pathology review, the original breast tumor also contained a sizeable contingent of cells expressing neuroendocrine markers, chromogranin A and synaptophysin.

Follow-up MRI of the spine and positron emission tomography-computed tomography (PET-CT) were performed one month after the end of SBRT. There was no evidence of loco-regional recurrence or new distant metastases. The patient continued letrozole in the following months and was also started on denosumab. A follow-up MRI five months post-SBRT demonstrated a recurrence with pathologic fracture at the level of L3. The patient underwent L3 corpectomy with fusion from L2-L4. The pathology report demonstrated intra-osseous and epidural tumor cells with neuroendocrine differentiation that also expressed the estrogen and progesterone receptors. The patient subsequently received post-operative SBRT (30 Gy in 4 fractions) to the spine. The patient has since been transferred to a community hospital for palliative chemotherapy. 

## Discussion

Breast carcinomas with neuroendocrine differentiation are generally regarded as rare tumors that represent a small percentage of all breast cancers. However, a recent analysis by Wachter, et al. suggested that approximately 20% of luminal B-like breast carcinomas exhibit neuroendocrine differentiation [7]. Although data in the literature is scarce, invasive neuroendocrine carcinoma of the breast appears to be an aggressive breast cancer subtype with higher metastatic rates and shorter overall survival (OS) than invasive mammary carcinomas not otherwise specified (IMC-NOS), as found in an analysis of the surveillance, epidemiology, and end results (SEER) database [8].

Although several case reports described the development of neuroendocrine cancers in Lynch syndrome patients, there is no known association between Lynch syndrome and a predilection in developing neuroendocrine breast carcinomas as opposed to other breast cancer subtypes [9]. A search within the cancer genome atlas breast invasive carcinoma (TCGA-BRCA) database suggests that 3.2% of the samples (N=963) harbored missense mutations or deletions in mismatch repair genes: mutS homolog 2 (MSH2), mutL homolog 1 (MLH1), mutS homolog 6 (MSH6), postmeiotic segregation increased 2 (PMS2) or epithelial cell adhesion molecule (EpCAM) [10].

Regarding the workup for early stage breast cancer, routine systemic staging is generally not indicated for clinical stage I-IIB breast cancer. Therefore, a bone scan would only have been indicated if the patient was symptomatic upon initial presentation. Furthermore, a bone scan was not an obligatory part of the inclusion criteria in the recently published prospective TAILORx trial [6]. Based on the staging exams and pathology, this patient’s profile fits with the indicated use of Oncotype DX testing.

The TAILORx trial has demonstrated a good correlation between tumor RS categorization and a patient’s risk of developing distant recurrences. Nevertheless, the validity of the assay in prognosticating and predicting patients with neuroendocrine features within their cancers is unknown and has not been specifically studied. Due to the perceived rarity of neuroendocrine breast carcinomas, immunohistochemical testing for neuroendocrine markers is not standard and may contribute to its underestimation especially within tumors of the molecular luminal B subgroup. As such, immunohistochemical stains for chromogranin A and synaptophysin could be added to institutional protocols or by the genomic assay laboratory prior to proceeding with further molecular testing for which the importance of neuroendocrine differentiation and components is unknown.

The rapid development of metastatic disease after diagnosis in this patient likely suggests the presence of synchronous occult metastatic disease and would have therefore excluded the use of a genomic test. The correct identification of a neuroendocrine breast carcinoma at diagnosis might have encouraged the treating physician to recommend additional staging exams such as a bone scan to rule out asymptomatic distant metastases from a potentially more aggressive cancer subtype and/or ruled out the use of Oncotype DX.

Despite recent advances, our understanding of how to integrate clinical parameters with molecular tools remains partial. Akin to clinical prognostic factors, current molecular assays have improved oncologists’ precision in discriminating patients’ risk of relapse and/or need for more aggressive therapies. Existing molecular assays are imperfect and bound to miss on exceptional cases. This situation is analogous to how computed tomography (CT) led to the development of high precision radiotherapy, it subsequently highlighted the importance of being accurate in delineating and tracking the movement of tumors to avoid missing the target. 

Currently, most genome-based assays only examine a small portion of the cancer genome. As whole genome next-generation sequencing (NGS) becomes more routine, this technology has the potential to provide the full picture of a specific type of cancer and revolutionize our approach to breast cancer treatment. Several recently launched research initiatives will help refine the role of gene sequencing in personalized medicine. One ambitious collaborative effort is the American Association for Cancer Research’s (AACR) project Genomics, Evidence, Neoplasia, Information, Exchange (GENIE), an international data-sharing project that will pool existing and future NGS data with longitudinal clinical outcomes and related pathology reports from multiple institutions worldwide.

Correspondingly, the ongoing National Cancer Institute (NCI) Molecular Analysis for Therapy Choice (NCI-MATCH) trial sequences 143 genes from patients’ tumors to determine whether they contain actionable genetic abnormalities matching an existing agent’s molecular target to determine whether this genome-based treatment strategy shows promise of effectiveness. Another example is the AURORA initiative for metastatic breast cancer. In this multinational metastatic breast cancer molecular profiling program, patients will donate archived primary tumor tissue as well as blood and prospectively collected biopsy material of metastatic lesions. The tumor tissue and the blood sample will then be subjected to NGS for a panel of cancer-related genes. The patients will be treated at the discretion of their treating physicians; however, depending on the molecular profiles found, patients may be directed to clinical trials for assessing molecularly targeted agents. 

## Conclusions

In conclusion, this case highlights that gene assays should not be used in the presence of a rare histological subtype such as neuroendocrine differentiated breast cancer. The addition of specific immunohistochemical stains into the normal pathology workflow may help to identify these rare cases and better orient diagnostic and treatment decisions. Finally, advances in gene sequencing may allow for a more complete picture of the cancer genome and will likely usher in a new era of genome-informed personalized medicine.
